# Cancer non-stem cells as a potent regulator of tumor microenvironment: a lesson from chronic myeloid leukemia

**DOI:** 10.1186/s43556-021-00030-7

**Published:** 2021-03-10

**Authors:** Naofumi Mukaida, Yamato Tanabe, Tomohisa Baba

**Affiliations:** Division of Molecular Bioregulation, Cancer Research Institute, Kanazawa University, Kakuma-machi, Kanazawa, 920-1192 Japan

**Keywords:** Basophil, Cancer stem cell, Leukemia stem cell, Megakaryocyte, Tissue-resident stem cell

## Abstract

A limited subset of human leukemia cells has a self-renewal capacity and can propagate leukemia upon their transplantation into animals, and therefore, are named as leukemia stem cells, in the early 1990’s. Subsequently, cell subpopulations with similar characteristics were detected in various kinds of solid cancers and were denoted as cancer stem cells. Cancer stem cells are presently presumed to be crucially involved in malignant progression of solid cancer: chemoresitance, radioresistance, immune evasion, and metastasis. On the contrary, less attention has been paid to cancer non-stem cell population, which comprise most cancer cells in cancer tissues, due to the lack of suitable markers to discriminate cancer non-stem cells from cancer stem cells. Chronic myeloid leukemia stem cells generate a larger number of morphologically distinct non-stem cells. Moreover, accumulating evidence indicates that poor prognosis is associated with the increases in these non-stem cells including basophils and megakaryocytes. We will discuss the potential roles of cancer non-stem cells in fostering tumor microenvironment, by illustrating the roles of chronic myeloid leukemia non-stem cells including basophils and megakaryocytes in the pathogenesis of chronic myeloid leukemia, a typical malignant disorder arising from leukemic stem cells.

## Introduction

**Fig. 1:**
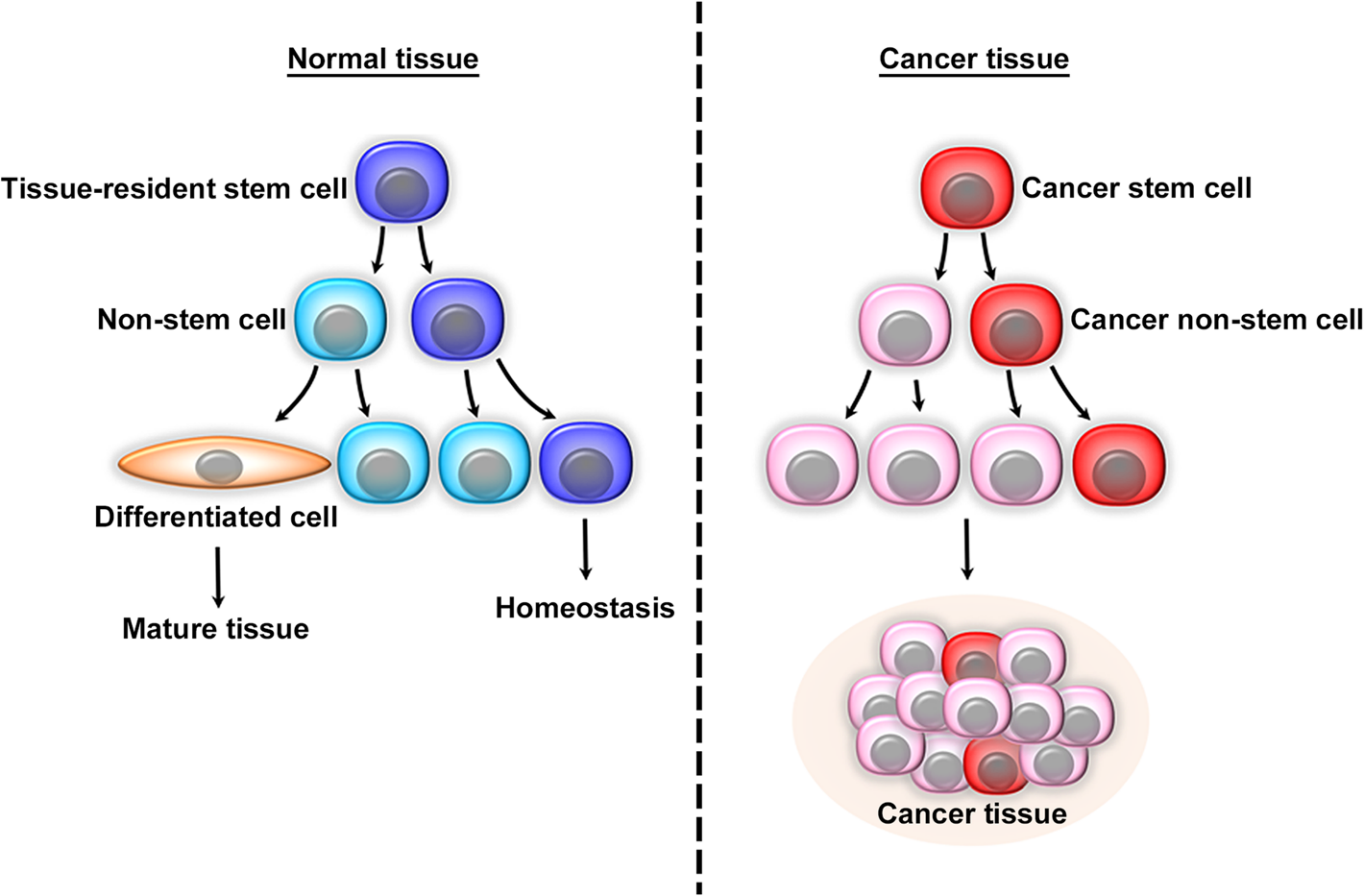
Hierarchy of stem and non-stem cells in normal and cancer tissues. Normal tissue-resident stem cells generate new stem cells through symmetric divisions (producing two similar stem cells) or asymmetric divisions (producing one stem and one non-stem cells). Stem cells self-renew to maintain homeostasis while non-stem cells lose self-renewal capacity and differentiate into mature cell types to exert normal tissue functions. Like normal tissue-resident tissue stem cells, CSCs generate a small number of CSCs with self-renewal capability and cancer non-stem cells, which predominate cancer tissues

Pluripotent stem cells in embryo can self-renew and can generate all mature cell types in the body as their potency to self-renew progressively decreases [1]. Adult organs possess populations of tissue-resident stem cells, which are capable of self-renewal to differentiate into all types of cells in the corresponding tissue [2]. Tissue-resident stem cells can generate new stem cells through symmetric divisions (producing two similar stem cells) or asymmetric divisions (producing a stem cell and a non-stem cell) (Fig. 1) [3]. The resultant tissue-resident cells can be sustained by interacting with their microenvironment, niche, through activation of various signaling pathways, particularly Wnt/β-catenin, Hedghog, and Notch pathways [2]. Simultaneously, non-stem cells lose self-renewal capacity and differentiate through a progenitor stage with a restricted differentiation capacity, to mature cell types, which are specific to their tissue of origin [2, 3]. Thus, tissue-resident stem cells are crucial for tissue homeostasis maintenance under both physiological and pathological conditions.

A seminal study reported the presence of a minor fraction of leukemia cells which can in vitro continue to proliferate similarly as hematopoietic stem cells (HSCs) can [4]. In 1990’s, evidence is accumulating to indicate that these cells have a self-renewal capacity and can propagate leukemia upon their serial transplantation into animals [5, 6]. Based on these properties, they are named as leukemia-initiating cells or leukemia stem cells (LSCs) [7]. Subsequently, cell subpopulations with similar characteristics were detected in various kinds of solid cancers including breast [8], brain [9, 10], colorectal [11], hepatocellular [12], and pancreatic cancers [13], and melanomas [14], and have been termed as cancer-initiating cells or cancer stem cells (CSCs). Like normal tissue-resident stem cells, LSCs and CSCs have a self-renewal ability to generate new stem cells through symmetric or asymmetric divisions [15, 16]. Self-renewal capacity of CSCs is maintained by the activation of several signaling pathways used by tissue-resident stem cells, such as Wnt/β-catenin [17, 18], Hedgehog [19], or Notch pathway [20], in a cell context-dependent manner. CSCs are presumed to be crucially involved in various carcinogenesis steps, particularly malignant progression [15, 16]. In addition to CSCs, asymmetric divisions simultaneously generate cancer non-stem cell populations which compose most of cancer cells present in cancer tissues (Fig. 1), but the roles of cancer non-stem cells in carcinogenesis are often overlooked.

In this review, we will briefly summarize biological aspects of CSCs and will discuss the potential roles of cancer non-stem cells in tumor microenvironment formation, by delineating the roles of non-stem cells in the pathogenesis of chronic myeloid leukemia (CML), a typical malignant disorder arising from LSCs.

## Properties of Cancer stem cells (CSCs)

### Cellular origin of CSCs

**Fig. 2:**
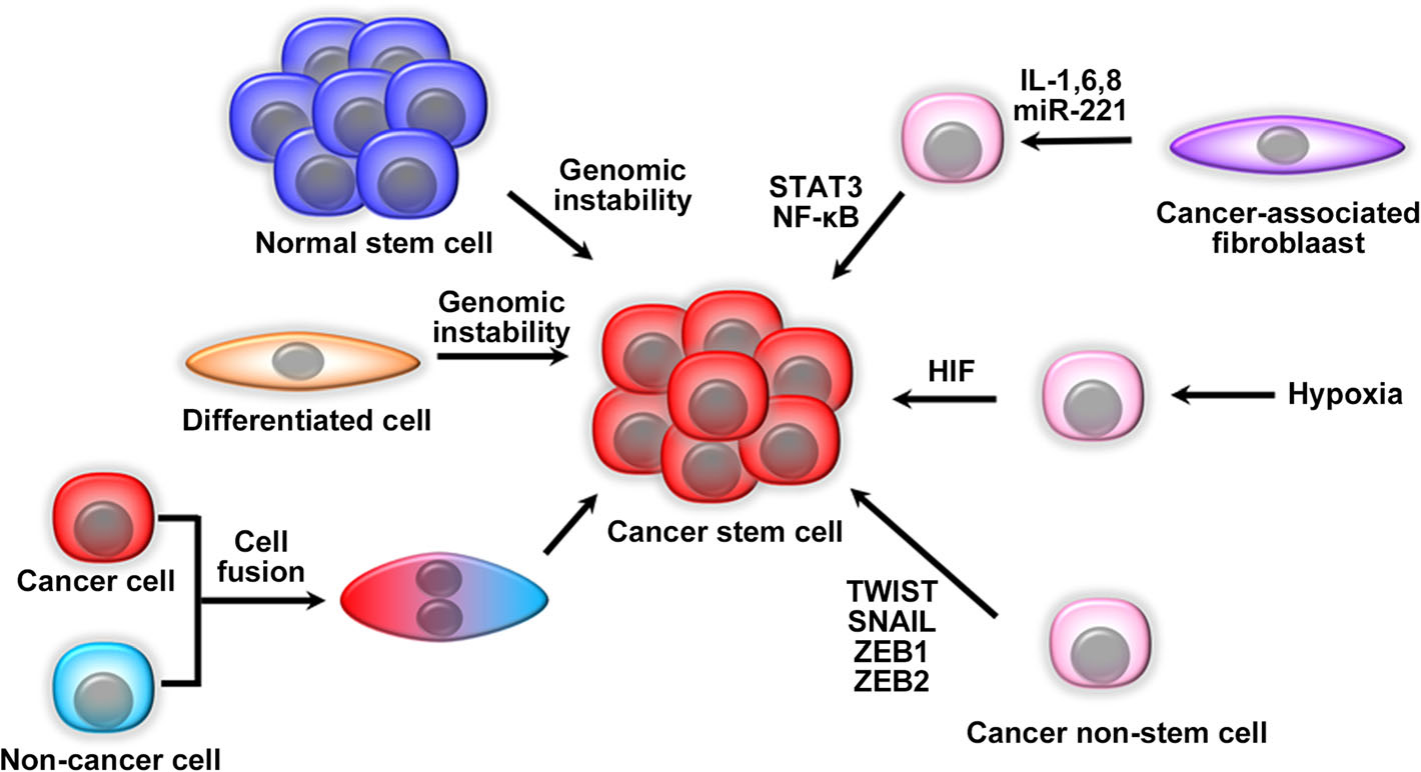
The origin of CSCs. Genetic mutations generate CSCs from tissue resident stem cells or differentiated cells. CSCs can be generated also by cell fusion between normal and cancer cells. Hypoxia induces cancer non-stem cells to display CSC phenotypes by activating hypoxia inducible factor (HIF) or signal transducer and activator of transcription (STAT)3 pathway. Moreover, the interaction between cancer and stromal cells produces various cytokines to activate STAT3/nuclear factor (NF)-κB pathways in both cancer and stromal cells, thereby driving CSC generation from non-CSC populations. Furthermore, CSCs are generated from non-CSC population by the action of a number of EMT-inducing transcription factors such as TWIST, SNAIL, ZEB1, and ZEB2

CSCs show very similar phenotypes to tissue-resident normal stem cells, and indeed, in several types of cancers, tissue-resident stem cells are the origin of CSCs (Fig. 2). For example, crypt stem cells are the origin of intestinal cancer [21] while skin basal cancer can arise from hair follicle stem cells [22]. This may arise from the conversion of normal stem cells to CSCs by random mutation accumulation during DNA replication [23]. Moreover, oncogenic *BCR-ABL* can transform hematopoietic stem cells into LSCs, and eventually can induce CML. On the contrary, other leukemogenic fusion genes, such as *MLL-ENL* [24] or the *MOZ-TIF* gene [25] can convert more committed hematopoietic progenitor cells (HPCs) into LSCs of acute myeloid leukemia (AML). Similarly, PIK3CA^H1047R^, the most frequent mutation observed in human breast cancer, can dedifferentiate lineage-committed cells of adult mouse mammary glands into multipotent CSC-like cells [26]. Thus, CSCs are derived from differentiated cells as well as tissue-resident multipotent stem cells (Fig. 2).

Accumulating evidence indicates that cancer non-stem cells can acquire CSC-like phenotypes under various conditions (Fig. 2). Genomic instability induces cancer non-stem cells to acquire CSC-like phenotypes, when the instability is enhanced by various causes including DNA damages induced by ultraviolet and mitomycin C, overexpression of a key regulator of cell cycle, Mad2, inhibition of an important kinase in mitosis such as Aurora B, or a key E3 ligase in cell cycle such as Cdh1 [27]. These events account for genomic instability-induced generation of CSCs from cancer non-stem cells, particularly at tumor recurrence after anti-cancer treatment. Injury to a tissue can enhance spontaneous fusion of tissue resident stem cells with non-stem cells, which can generate new hybrid pluripotent cells [28]. It was proposed that similar cell-cell fusion events can occur among cancer cells or between cancer cells and normal cells, and can eventually create CSCs [29]. This assumption is substantiated by the observations that human breast cancer cells acquired CSC-like phenotypes through the fusion with normal breast epithelial cells [30] or adipose tissue-derived stem cells [31]. Normal resident cells present in tumor microenvironment can maintain CSC phenotypes by secreting various mediators. Endothelial cells can sustain CSCs by secreting interleukin (IL)-6 [32, 33] or basic fibroblast growth factor [34], while fibroblasts can maintain CSC phenotypes by secreting soluble mediators such as a chemokine, CCL2 [35, 36].

The activation of transcription factors can trigger CSC generation from non-CSC population (Fig. 2). Hypoxia induces cancer non-stem cells to exhibit CSC-like phenotypes by activating hypoxia-inducible factor [37] or signal transducer and activator of transcription (STAT)3 pathway [38]. IL-6-STAT3 pathway activation induced cancer-associated fibroblasts (CAFs) to generate microvesicles containing miR-221, which was horizontally transferred to breast cancer non-stem cells, thereby promoting their acquisition of CSC-like phenotypes, particularly in combination with hormone therapy [39]. Additionally, cancer and stromal cells produce various inflammatory cytokines, such as IL-1, IL-6, and IL-8, which in turn activate STAT3/NF-κB pathways in both cancer and stromal cells [40]. Activation of these pathways further enhances cytokine production and eventually forms a positive feedback loops that in turn drive CSC generation from cancer non-stem cells and their self-renewal. Additionally, as CSC generation from cancer non-stem cells can be promoted by a number of transcription factors including TWIST, SNAIL, ZEB1, and ZEB2, the transcription factors that regulate epithelial-mesenchymal transition (EMT), CSC generation is accompanied by EMT [41, 42], which can provide epithelium-derived cancer cells with mesenchymal phenotypes including a motile capacity to exit from the primary sites [43]. Thus, CSCs are presumed to be prone to metastasize due to their mesenchymal phenotypes [43].

### Phenotypes of CSCs

CSCs are defined functionally as a cancer cell with a capacity to develop tumor upon its serial transplantation into an animal or to form *in vitro* spheres upon serial passages [15, 16]. However, both assays are time-consuming and lack reproducibility as exemplified by differences in tumorigenic capacity according to the types of transplanted animals [14]. Thus, a vast number of studies have been conducted to identify surface markers, which are expressed selectively by CSCs (Table 1) [44]. CD44 is a transmembrane glycoprotein, which arises from a single gene which in humans contains 19 exons, but its alternative splicing generates variant CD44 isoforms (CD44v) as well as the standard form of CD44 (CD44s) [45]. CD44 is expressed by CSCs in various types of cancers including breast, ovary, prostate, and pancreas cancer, head and neck squamous cell cancers, and can sustain stemness by interacting with hyaluronan present in CSC niche [47]. Another notable surface marker of CSCs is CD133 [46], which is a five transmembrane glycoprotein consisting of two large extracellular loops and two small cysteine-rich intracellular loops [48]. CD133 is expressed also by CSCs in various types of cancers including breast, ovary, prostate, colon, liver, lung, and renal cancers, glioblastoma, and medulloblastoma, but it still remains elusive on the roles of CD133 in CSC maintenance.

**Table 1:** Representative surface markers to identify CSCs in various types of cancers

Surface marker	Cancer type	Reference
CD15	glioblastoma, medulloblastoma	[44]
CD24	breast, liver, colon, gastric cancer	[44]
CD33	acute myeloid leukemia	[44]
CD44	breast, ovary, prostate and pancreas cancer, head and neck squamous cell carcinoma	[45]
CD123	acute myeloid leukemia	[44]
CD133	breast, ovary, prostate, colon, liver, lung, and renal cancer, glioblastoma, medulloblastoma	[46]
CD166	lung, colon cancer	[44]

Aldehyde dehydrogenase (ALDH)1, a member of ALDH family, is a detoxifying enzyme responsible for intracellular aldehyde oxidation [49] and can induce the differentiation of stem cells by oxidizing retinol to retinoic acid [50]. ALDH1 is abundantly expressed in CSCs of breast [51], ovarian [52] and colorectal cancers [53]. ALDH1-expressing cells can be identified by using flow cytometry-based Aldefluor assay, which relies on a capacity of ALDH1 to convert a non-fluorescent molecule to a fluorescent product [54]. An additional flow cytometrical method is used to detect CSCs, based on their augmented expression of ABC transporters including ATP-binding cassette subfamily-B member 1 (ABCB1) and ATP-binding cassette subfamily-G member 2 (ABCG2) [55]. With these transporters, CSCs can eject out a fluorescent dye, Hoechst 33342, with a high efflux efficiency and appear as a negatively-stained population or side population (SP) on a flow cytometry [56].

Until present, no single molecules, however, have been identified as a specific marker for CSCs. Moreover, even with the combined use of several markers, the proportions of CSCs among total cancer cells are frequently estimated to be high in solid cancers, reaching higher than 10 %. Considering that normal tissue-resident stem cells comprise less than 1 % of total cells, it is highly likely that hitherto identified CSCs contain non-CSC population in solid cancers. Thus, identification of specific markers is required to discriminate CSC from non-CSC populations and to elucidate the functions of CSCs and cancer non-stem cells in more detail.

### Pathological roles of CSCs

**Fig. 3:**
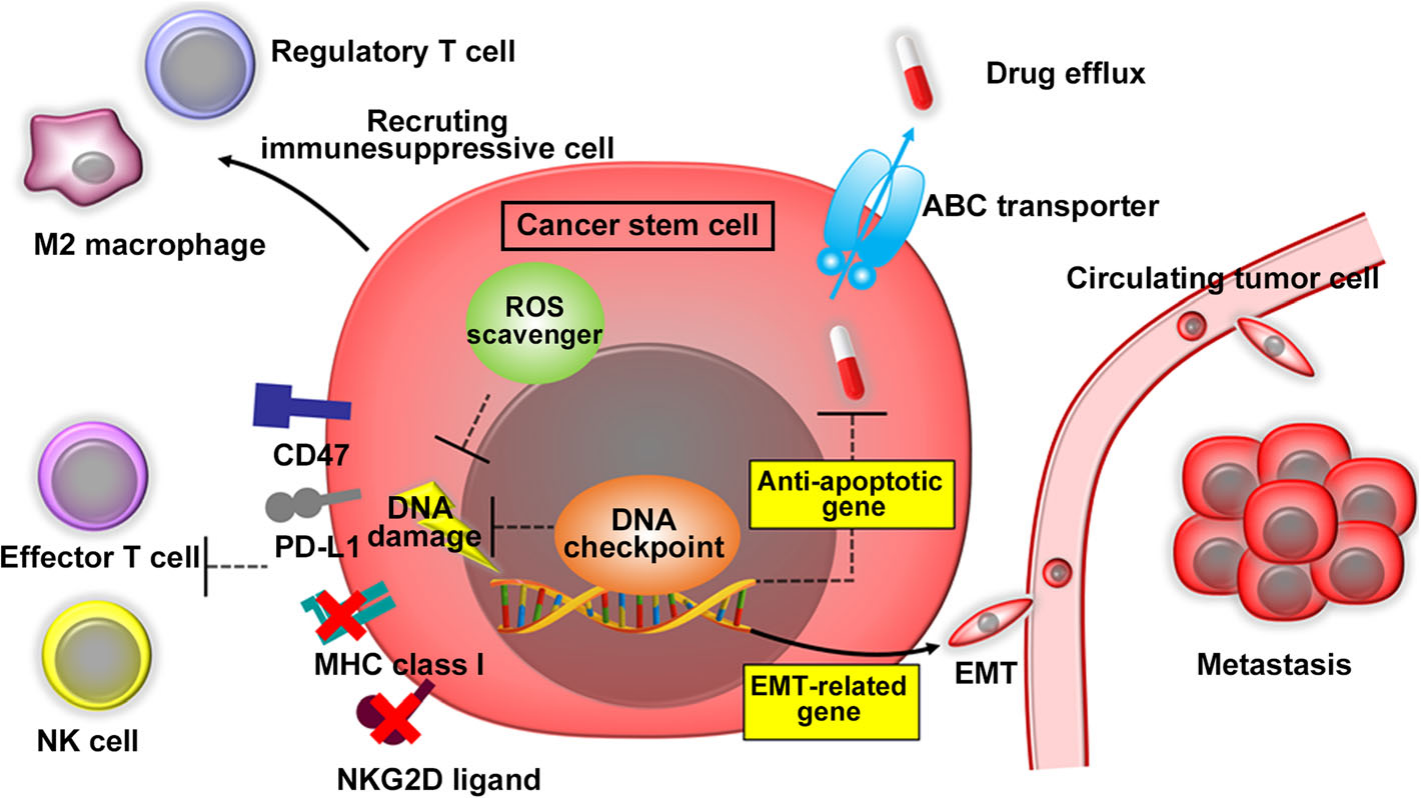
Presumed biological properties of CSCs. CSCs are in a dormant state and therefore, are resistance to chemotherapy and irradiation, which are mainly effective against proliferating cells. Moreover, CSCs exhibit enhanced expression of ABC transporters and anti-apoptotic molecules, efficiently expel chemotherapeutic drugs and to counteract drug-induced apoptosis, respectively. Furthermore, CSCs display higher levels of reactive oxygen species (ROS) scavengers and augmented DNA checkpoint activation, to counteract ROS-induced adverse effects and to prevent DNA damages, respectively. CSCs dampen tumor immunity by inhibiting effector T cell and inducing immunosuppressive M2-macrophages and regulatory T cells (Tregs). Moreover, CSCs exhibit enhanced expression of immune checkpoint molecules to hamper T cell-mediated tumor immunity. CSCs further express reduced expression of HLA class I antigen and KKG2D ligand to escape cytotoxicity mediated by effector T cells and natural killer (NK) cells, respectively. Furthermore, CSCs display EMT phenotypes, which are indispensable for metastasis processes

Evidence is accumulating to indicate the crucial involvement of CSCs in various aspects of malignant progression of cancer cells, including resistance to therapy [57], immune evasion [58], and metastasis [59]. Several mechanisms are presumed to account for CSC-mediated resistance to anti-cancer therapies (Fig. 3). In order to maintain tissue homeostasis, adult tissue-resident stem cells are in a state of cellular dormancy, where cells are recruited into G_0_ phase but remain capable of cell division in response to mitotic stimuli [60]. CSCs are also in a cellular dormant state and as a consequence, are resistance to chemotherapy and irradiation, which are mainly effective against proliferating cells [61]. However, as non-CSC population can also be moved into a dormant state [62], non-CSC population can also contribute to resistance to anti-cancer therapy. Additionally, most anti-cancer treatments can induce apoptosis in cancer cells [63] but CSCs in prostate and breast cancer exhibit enhanced expression of a potent anti-apoptotic molecule, B-cell lymphoma 2 (Bcl-2) [64, 65], thereby counteracting drug-induced apoptosis. Moreover, CSCs express higher levels of ABC transporters including ABCB1 and ABCG2, than non-CSC population [55]. These transporters can efficiently expel a wide variety of chemotherapeutic drugs including alkylating agents, antimetabolites, topoisomerase inhibitors, taxanes, and vinca alkaloids [66] and eventually endow CSCs with resistance to these drugs. Furthermore, normal tissue-resident stem cells and CSCs express higher levels of reactive oxygen species (ROS) scavengers, such as glutathione biogenesis synthesis genes, thereby preventing ROS-induced DNA damages upon irradiation [67]. Glioma CSCs exhibit radioresistance by augmenting DNA checkpoint activation as evidenced by increased phosphorylation of the ataxia-telangiectasia-mutated (ATM), checkpoint 1 (Chk1), and Chk2 [68].

CSCs are equipped with an ability to suppress the recognition by innate and adaptive immunity and to reshape tumor microenvironment into an immunosuppressive one (Fig. 3). CSCs recruit macrophages and induce their polarization towards M2 macrophage with a capacity to inhibit immune response [69]. Melanoma and glioma CSCs can inhibit effector T cell activation and simultaneously can induce regulatory T cells (Treg), thereby counteracting specific tumor immunity [70, 71]. Moreover, compared with non-CSC population, CSCs in breast and lung cancers express higher levels of immune checkpoint molecules including PD-L1 [72] and CD47 [73], respectively. PD-L1 and CD47 can inhibit the activities of activated T cells and macrophages, respectively, thereby hampering tumor immunity. Furthermore, lung cancer CSCs and AML LSCs exhibit reduced expression of HLA class I antigen [74] and NKG2D ligand [75], respectively. Decreased HLA class I antigen and NKG2D expression can constrain cytotoxicity mediated by effector T cells and natural killer cells, respectively.

CSCs are presumed to contribute also to metastasis process. EMT provides cancer cells with mobile mesenchymal cell-like phenotypes, which can facilitate their exit from the primary sites and therefore, is an essential step for metastasis [43]. As EMT can induce cancer cells to express CSC phenotypes [41, 42], CSCs exhibit EMT phenotypes, which are presumed to have crucial roles in metastasis [76]. Moreover, circulating tumor cells have an ability to metastasize to distant organs and exhibit specific changes in DNA methylation that are shared by CSCs [77]. However, more detailed studies are necessary to elucidate the roles of CSCs in metastasis.

CSCs have presently been an intensive focus of cancer research due to their crucial involvement in malignant progression processes such as therapy resistance, immune evasion, and metastasis, whereas less attention is paid to cancer non-stem cells, which compose most cancer cells in cancer tissues. The study on cancer non-stem cells meets with difficulties due to the shared functional and phenotypic characteristics between CSCs and cancer non-stem cells in solid cancer. In CML, however, non-stem cells, major components of leukemia cells, are phenotypically distinct from stem cells [78]. Thus, CML can be a good subject to study the roles of non-stem cells in carcinogenesis. In the following sections, we will discuss the potential roles of non-stem cells in CML pathogenesis, in order to facilitate the understanding of the roles of cancer non-stem cells in solid tumor progression.

## Chronic myeloid leukemia (CML)

### Leukemia stem cells (LSCs) in CML

**Fig. 4:**
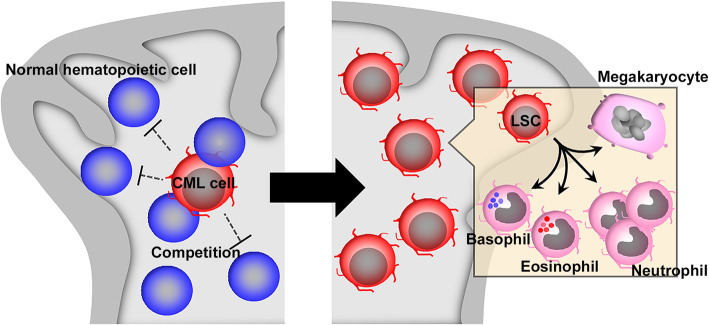
CML progression. *BCR-ABL* gene transforms HSCs into LSCs, which gradually and clonally proliferate in competition with a large number of normal hematopoietic cells to eventually occupy bone marrow and migrate into other tissues including peripheral blood and spleen. CML LSCs simultaneously differentiate into more mature leukemia non-stem cells and as a consequence, CML cells consist of a small number of LSCs with the majority of leukemia non-stem cells including neutrophils, eosinophils, basophils and megakaryocytes. Moreover, these differentiated cells morphologically exhibit distinct features from LSCs but similar characteristics with normal counterparts

CML harbors a characteristic abnormal chromosome, the Philadelphia chromosome (Ph), which arises from a reciprocal translocation between the long arm of chromosome 9 (ch9) and 22 (ch22) [78]. This reciprocal translocation replaces the upstream control element of the *ABL-1* gene, the human analogue of the *v-ABL* oncogene, with the *BCR* gene and generates *BCR-ABL* fusion gene, which encodes a constitutively activating tyrosine kinase with a capacity to phosphorylate various substrates including molecules involved in cell proliferation [79]. As a consequence, *BCR-ABL*-expressing hematopoietic cells can proliferate as LSCs to induce pathological changes observed in CML [80, 81] (Fig. 4). Moreover, the transduction of *BCR-ABL* gene conferred the capacities to self-renew in vitro and to cause leukemogenesis on murine HSCs but not HPCs [25] in contrast to acute myeloid leukemia models, where leukemia can develop upon the transduction into HPCs of oncogenic fusion genes such as the *MLL-ENL* [24] or the *MOZ-TIF* gene [25]. Thus, it is probable that CML LSCs are derived from BCR-ABL-transformed HSCs. However, a single *BCR-ABL* copy expressed from endogenous *BCR* locus, enhanced bone marrow grafting capacity without inducing any neoplasm [82]. Thus, BCR-ABL is prerequisite but not sufficient for CML LSC generation, which might additionally require *BCR-ABL* copy number amplification, secondary mutations and/or genomic instability [78].

**Fig. 5:**
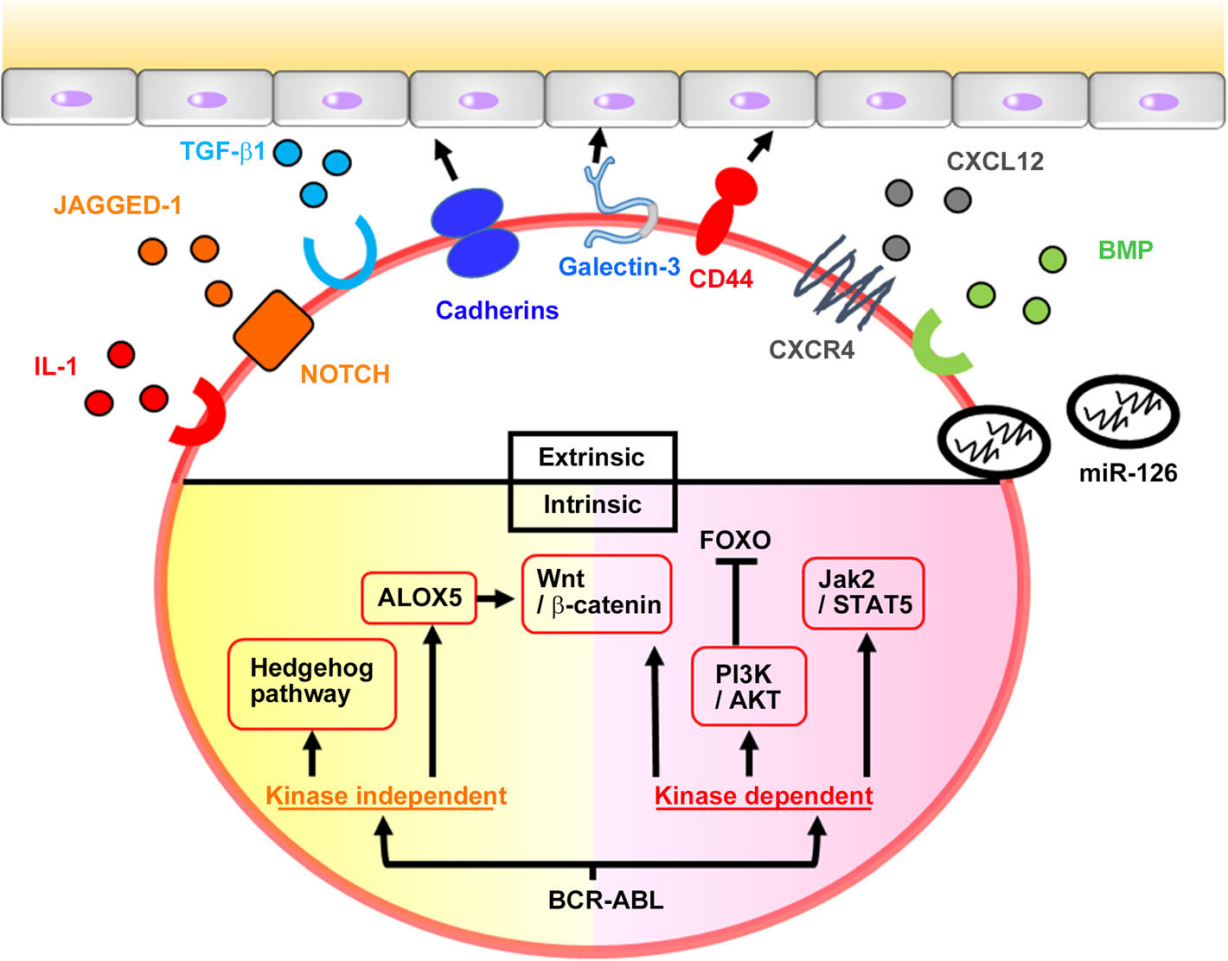
Presumed mechanisms involved in CML LSC maintenance. Phosphoinositide 3-kinase (PI3K)/Akt/FOXO axis [89], Wnt signaling [90, 91], and janus kinase (JAK)2/ signal transducer and activator of transcription (STAT)5 signaling [92], are directly activated by kinase activity of BCR-ABL and sustain LSC survival. Moreover, activation of Hedgehog signaling [93] and ALOX5 [94] can maintain LSC survival independently of BCR-ABL kinase activity. LSC survival further requires their localization to bone marrow niche with the help of several LSC-expressing adhesion molecules such as cadherins [95], CD44 [96], and galectin-3 [97]. Furthermore, bone marrow resident cells secrete various mediators including Jagged 1 (a NOTCH ligand) [98], transforming growth factor (TGF)-β1 [99], bone morphogenic proteins (BMPs) [100], a chemokine, CXCL12 [101], interleukin (IL)-1 [102], and exosomes containing miR-126 [103] to sustain the stemness of CML LSCs

In CML, LSCs are presumed to reside within lineage marker (Lin)^-^CD34^+^CD38^-^ fraction of leukemia cells [83, 84], but this phenotype is shared by normal HSCs [85]. Subsequent studies identified IL-2 receptor-α (CD25) [86], dipeptidyl peptidase 4 (DPP4, CD26) [85], Siglec-3 (CD33) [85], scavenger receptor-B2 (SR-B2, CD36) [85, 87], and IL-1 receptor accessory protein (IL-1RAP) [85, 88] as surface markers expressed predominantly by CML LSCs (Table 2). Several mechanisms are presumed to be involved in CML LSC maintenance and survival (Fig. 5). Phosphoinositide 3-kinase (PI3K)/Akt/FOXO axis [89], Wnt signaling [90, 91], and Janus kinase (JAK)2/STAT5 signaling [92], are directly activated by kinase activity of BCR-ABL and sustain LSC survival. Moreover, frequent failure of BCR-ABL kinase inhibitors to eradicate completely CML LSCs suggests the involvement of BCR-ABL kinase-independent intrinsic pathways in LSC maintenance. Indeed, activation of Hedgehog signaling [93] and ALOX5 [94] can sustain LSC survival independently of BCR-ABL kinase activity. LSC survival further requires their localization to bone marrow niche with the help of several LSC-expressing adhesion molecules such as cadherins [95], CD44 [96], and galectin-3 [97]. Furthermore, bone marrow resident cells maintain CML LSCs by secreting various soluble factors such as Jagged 1 (a NOTCH ligand) [98], transforming growth factor (TGF)-β1 [99], bone morphogenic proteins (BMPs) [100], a chemokine, CXCL12 [101], IL-1 [102], and exosomes containing miR-126 [103].

**Table 2:** Expression pattern of surface markers on normal and CML CD34^+^CD38^-^ and CD34^+^CD38^+^ cell populations

Marker	CML CD34^+^CD38^-^	Normal CD34^+^CD38^-^	CML CD34^+^CD38^+^	Normal CD34^+^CD38^+^	
IL-2 receptor **α** (CD25)	++	-	+/-	+/-	[86]
Dipeptidyl peptidase 4 (DPP4, CD26)	++	-	+/-	-	[85]
Siglec-3 (CD33)	++	-	+	++	[85]
scavenger receptor-B2 (SR-B2, CD36)	++	+/-	++	++	[85, 87]
IL-1 receptor accessory protein (IL-1RAP)	+	-	+	+	[85, 88]

### Clinical aspects of CML

CML has three distinct clinical phases: chronic phase (CP), accelerated phase (AP), and blast phase (BP) [104]. At diagnosis, most CML patients are in CP, which is characterized by increases in neutrophil, eosinophil and basophil numbers in peripheral blood and bone marrow, with the predominance of mature leukocytes over blasts (usually less than 5 %). As the origin of CML LSCs is HSCs, a minor population among hematopoietic cells [25], BCR-ABL generates only a limited number of LSCs. LSCs appear in bone marrow in a small number and proliferate clonally in competition with a large number of normal hematopoietic cells. Thereafter, LSCs occupy bone marrow and eventually migrate into other tissues including peripheral blood and spleen (Fig. 4). As CML LSCs still share a differentiation ability with normal HSCs, CP-CML LSCs simultaneously differentiate into various morphologically mature but molecularly malignant BCR-ABL-expressing leukocytes. Thus, the leukocytes in CP are heterogeneous, consisting of a small number of LSCs with the majority of leukemia non-stem cells (Fig. 4). Nonetheless, tyrosine kinase inhibitors (TKIs) targeting BCR-ABL can efficiently eradicate most BCR-ABL-expressing CP-CML cells and as a consequence, have drastically improved the prognosis of CP-CML patients since their clinical introduction two decades ago [104].

BP is defined as the state of an increased proportion of blasts (usually more than 20 %) in blood or bone marrow and is also called as blast crisis, while AP is an intermediate phase between CP and BP, with resistance to TKIs and less blasts (less than 20 %) in blood or bone marrow [105]. Thus, BP is generally a late feature of progression but is also observed in a small proportion of newly diagnosed CML patients [106]. Evidence is accumulating to indicate that LSCs in CP may evolve into those in BP through several molecular changes. BCR/ABL affects the efficiency and fidelity of major DNA double strand breaks (DSBs) repair mechanisms by stimulating WRN (mutated in Werner syndrome), thereby facilitating genomic instability [107]. Genomic instability predisposes to additional gene mutations, which are observed in BP-CML cells, such as *p16* deletion [108], *p53* loss of function [109], loss of retinoblastoma gene product [110], increased *Evi-1* expression [111], or mutations in *RUNX-1*, *ASXL1*, and *isocitrate dehydrogenases* [112]. Moreover, aberrant histone deacetylase activities induce epigenetic dysregulation, thereby conferring more aggressive phenotypes on cells in BP [113]. Musashi2, an oncogenic RNA binding protein, is aberrantly expressed in BP leukemia cells and is physically associated with the transcript of BCAT1, a cytosolic aminotransferase for branched-chain amino acids to enhance its expression and to eventually promote cell proliferation [114]. Intrinsic refractoriness of AP-blast cells to TKIs commonly necessitates additional chemotherapeutic agents, which are mostly ineffective [106].

TKI treatment can keep CP-CML patients under favorable long-term disease control with about 90 % overall survival, 90 % complete cytogenetic response, and 90 % major molecular response which was defined as a reduction of at least 3 log in the BCR-ABL1 value from the standardized baseline level on the International Scale, at 10 years after the treatment initiation [115]. This incited the attempt to discontinue TKI treatment in CP-CML patients in 2-year long molecular remission defined as *BCR–ABL*/*ABL* levels lower than a detection threshold corresponded to a 5-log reduction [116]. This first study demonstrated that approximately 40 % CP-CML patients did not relapse until 1 year after the discontinuation of a TKI, imatinib, and that all relapsing patients responded well to the reintroduction of imatinib. These observations were validated by a subsequent meta-analysis on CML patients who underwent imatinib termination [117]. Moreover, a large-scale multi-center trial demonstrated that molecular relapse-free survival was 50 % at 24 months after the treatment discontinuation, in the patients who were treated with imatinib or second-line TKIs, and stayed for at least 1 year in deep molecular response, which was defined as less than 0.01 % *BCR-ABL* on the International Scale or undetectable *BCR-ABL* in samples with 10,000 or more *ABL* transcripts or 24,000 or more *GUS* transcripts [118].

Recurrence is presumed to arise from LSCs, which survive TKI treatment through several mechanisms [7]. Additional mutations in *BCR-ABL* gene can confer resistance to TKIs on LSCs [119]. Moreover, BCR-ABL induces ROS generation in CP-CML cells and eventually genomic instability, which can contribute to resistance to TKIs [120] as similarly observed on BP-CML cells. CP-CML LSCs can survive TKI treatment by activating BCR-ABL-kinase-independent pathways including Hedgehog signaling [93] and ALOX5 [94]. Furthermore, bone marrow resident cells confer TKI resistance on LSCs by either promoting the interaction of LSCs with bone marrow niche [95–97] or secreting stemness-maintaining mediators [98–103]. Nevertheless, the interaction with various types of cells present in bone marrow, is required for CML LSC maintenance.

Even in TKI era, a small number of CP-CML still progresses to AP-CML and eventually BP-CML [105]. Thus, it is necessary to identify and/or predict CP-CML patients who are at a high risk to develop AP-CML and/or BP-CML. Two scores were proposed to predict the prognosis of CP-CML patients based on the clinical findings at diagnosis [121, 122]. Both scores utilize four parameters: blast proportions and platelet counts in peripheral blood, age, and spleen size. Moreover, the World Health Organization proposed the criteria for AP-CML: basophilia (20 % more in peripheral blood), treatment-resistant persistent leukocytosis, splenomegaly and thrombocytosis, and increased proportions of blasts in peripheral blood and bone marrow [105]. Alternatively, increases in non-stem cells such as basophils and platelets are associated with poor prognosis of CML patients and therefore, it is highly likely that leukemia non-stem cells such as basophils and megakaryocytes, a precursor of platelets, can contribute to CML pathogenesis and progression. We will discuss this assumption in detail in the following sections.

### Basophils in CML pathogenesis

Basophils are the least abundant granulocytes in peripheral blood. Basophils and tissue-resident mast cells share many biological features including the presence of cytoplasmic basophilic granules, surface expression of high-affinity IgE receptor, and activation-induced release of chemical mediators, but they are distinct cell lineage differentiated from HSCs in bone marrow [123]. Human basophils develop from common basophil-eosinophil precursors, which differentiate from HSCs through the stage of common myeloid precursors [124]. In addition to basophil-eosinophil precursors, basophil-mast cell progenitors were identified in mouse but not human bone marrow, and a transcription factor, CCAAT/enhancer binding protein (C/EBP) α, determined the fate of precursors to basophil differentiation [125]. IL-3 and thymic stromal lymphopoietin (TSLP) can induce bone marrow progenitors to generate basophils with distinct gene signatures [126]. Basophils and basophil-committed colony-forming units are aberrantly produced in most CML patients [127], partly due to BCR-ABL-mediated C/EBPα activation [128]. Moreover, evidence is accumulating to indicate the association of basophilia with poor prognosis in CML patients in pre-TKI [129, 130] and post-TKI eras [131]. Several mechanisms were proposed to explain this association.

Vessel density is increased, together with tortuous vessel architecture and augmented branching in CML bone marrow [132] and was proposed to be an independent parameter to predict worse prognosis of CML patients [133]. Moreover, CML patients’ serum displayed enhanced levels of several angiogenic factors including vascular endothelial growth factor (VEGF) and hepatocyte growth factor (HGF) [134]. Human normal basophils contain VEGF in their secretory granules and can release it upon immunological activation [135]. BCR-ABL can induce VEGF expression in a mouse hematopoietic progenitor cell line [136], but it remains elusive on the capacity of basophil-like CML cells to secrete VEGF. Increased plasma HGF levels were associated with poor prognosis of the patients [133]. Basophil-like CML cells were identified to be a major cellular source of HGF, which could augment endothelial cell migration [137]. Additionally, basophil-like CML cells release abundantly tryptase [138], which is stored in their cytoplasm and can stimulate vascular tube formation [139]. Moreover, as tryptase is a potent mitogen for fibroblasts [140], it can contribute to the development of bone marrow fibrosis, which is associated with poor prognosis of CML patients [141] and their poor response to TKI treatment [142]. Thus, these basophil-derived mediators may be able to induce CML progression by affecting bone marrow microenvironment.

A characteristic feature of CML LSCs is their low ability to stay in bone marrow niche and their high capacity to redistribute to peripheral blood [143]. Their decreased remaining in bone marrow is associated with decreased availability of a chemokine, CXCL12, which can retain LSCs as well as normal HSCs [144]. CXCL12 can be degraded and inactivated by a surface enzyme, DPP4/CD26 [145], which is expressed by basophil-like CML cells [127] as well as CML LSCs [85]. Thus, CD26-mediated CXCL12 inactivation may account for LSC redistribution to peripheral blood. Their redistribution can be further facilitated by vascular permeability-enhancing mediators such as histamine, which is generated by the action of histidine decarboxylase expressed by basophil-like CML cells [146]. HGF can in vitro augment granulocyte-macrophage (GM)-colony forming unit (CFU) formation from CML blast cells, which express Met, a specific receptor for HGF [147]. However, the pathological relevance of this observation has not been yet determined in CML models or patients.

**Fig. 6:**
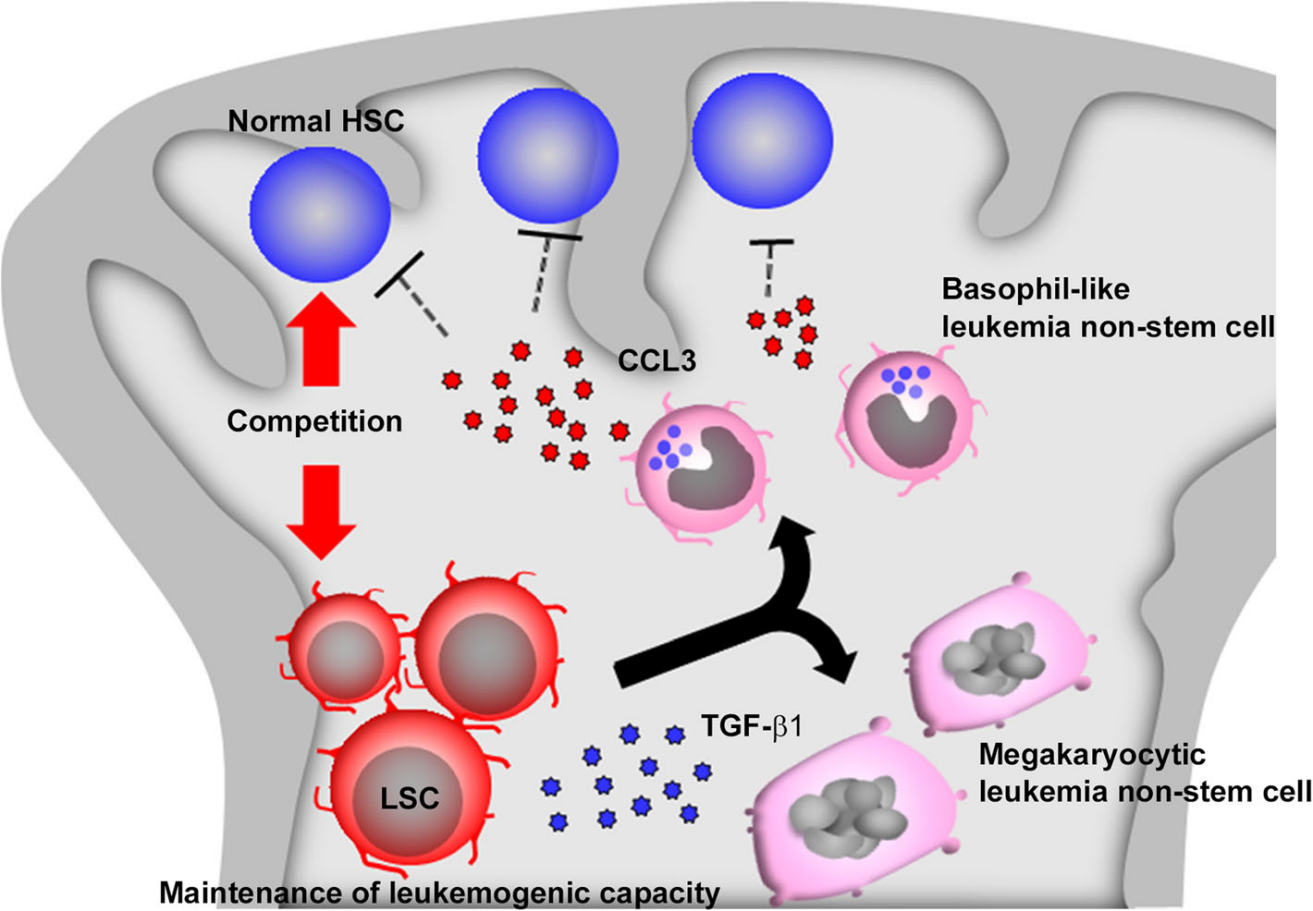
Presumed roles of basophil-like and megakaryocytic leukemia non-stem cells in CML pathogenesis. LSCs massively generate basophil-like leukemia non-stem cells, which abundantly produce a chemokine, CCL3. The produced CCL3 inhibits the functions of normal HSCs which are present in a large number especially at the early phase of CML bone marrow and as a consequence, gives advantage to LSC proliferation. Concomitantly, *BCR-ABL* gene expression induces the appearance of senescent megakaryocytic leukemia non-stem cells in bone marrow. These megakaryocytic leukemia non-stem cells abundantly produce TGF-β1, which can sustain leukemogenic capacity of LSCs in bone marrow, thereby promoting CML propagation

CCL3, previously known as macrophage inflammatory protein (MIP)-1α, can directly inhibit normal hematopoietic stem/progenitor cell (HSPC) proliferation [148, 149], through the interaction with its specific receptors, CCR1 or CCR5 [150]. The transplantation of *BCR-ABL*-expressing LSCs in vivo induced aberrant CCL3 expression in bone marrow [144]. Moreover, *ABL* gene conferred the resistance to CCL3 by abrogating CCL3-mediated intracellular calcium influx with few effects on its receptor expression [151]. These observations prompted us to investigate the role of endogenously-produced CCL3 in CML pathogenesis [152]. When BCR-ABL gene was transduced into CCL3-deficient mouse-derived HSPCs to generate CML LSCs, the resultant LSCs induced CML development in irradiated hematopoiesis-incompetent mice but failed to do in un-irradiated hematopoiesis-competent mice, which preserved normal hematopoietic cells in bone marrow. Moreover, admixture with CCR1- or CCR5-deficient HSPCs blunted the leukemogenic ability of wild-type mouse-derived LSCs [152], suggesting that leukemia cell-derived CCL3 acts mainly on CCR1- or CCR5-expressing normal hematopoietic cells in bone marrow to promote leukemogenesis. Given the differential effects of CCL3 on ABL-expressing and non-expressing cells [151], it is likely that CCL3 can dampen normal hematopoiesis and can reciprocally favor leukemogenic hematopoiesis (Fig. 6).

This notion has been further substantiated by our subsequent study [153]. CCL3 was expressed constitutively by normal basophils as well as basophil-like CML cells in mouse CML bone marrow, and negatively regulated normal hematopoietic process, particularly hematopoietic reconstitution after bone marrow transplantation. Depletion of basophil-like CML cells markedly reduced LSC numbers and eventually delayed CML progression [153]. Moreover, the administration of a CCR5 antagonist, maraviroc [154], prevented dramatically CML development when it was administered immediately after LSC transplantation but the effects were abrogated when administration started 2 weeks after LSC injection [153]. Thus, massively expanding basophil-like CML cells produce abundantly CCL3, which can inhibit normal hematopoiesis and can reciprocally facilitate CML LSC proliferation, particularly at the early phase, thereby advancing CML development (Fig. 6). As CCL3 was abundantly expressed by basophils in bone marrow of CML patients, it is probable that human leukemic basophils can contribute to LSC proliferation in CML.

### Megakaryocytes in CML pathogenesis

Platelets have diverse impacts on development and progression of solid tumors, particularly by accelerating tumor growth through angiogenesis induction and supporting tumor cells to evade the immune system and extravasate to metastatic organs [155]. However, until present, there are no reports on the precise roles of platelets in CML pathogenesis and progression [156].

Megakaryocytes in CML exhibit atypical features such as cytoplasmic vacuolation, smaller diameter, and hetergenous distribution of cytoplasmic granules [157]. Moreover, CML megakaryocytes displayed a shift towards lower ploidy number and about 60 % were less than 8N, compared with healthy volunteers showing the mean modal ploidy number of 16N [158, 159]. Of interest is that either interferon-α treatment in pre-TKI era [160] or TKI treatment [161] decreased small-sized megakaryocytes, together with improved cytogenetic response, suggesting that morphological abnormalities in megakaryocytes are closely associated with CML pathogenesis. Furthermore, in pediatric CML, increased megakaryocyte proliferation was associated with bone marrow fibrosis [162], an independent poor prognostic complication of CML [163], although it remains elusive how megakaryocyte promoted fibrosis in CML.

We recently observed that bone marrow transplantation of BCR-ABL-transduced LSCs induced massive accumulation of BCR-ABL-expressing megakaryocytes in bone marrow in CML model [164], similarly as observed on human CML patients [165]. Senescence can be induced in fibroblasts and epithelial cells by the activation of oncogenes such as Ras or B-Raf [166]. Likewise, we observed that senescence was provoked selectively in expanding megakaryocytes in CML by an oncogenic fusion protein, BCR-ABL, and was abrogated together with megakaryocyte reduction by deletion of p16 and p21 [164], the molecules crucially involved in senescence [167]. Thus, senescence may be required for megakaryocyte generation also in CML as well as that in normal hematopoiesis [168]. Senescence is frequently accompanied by senescence-associated phenotype (SASP) characterized by enhanced expression of several pro-inflammatory cytokines including IL-1, IL-6, CXCL8 and TGF-β1 [169] and indeed, senescent CML megakaryocytes expressed TGF-β1 in p16- and p21-dependent manner [164]. TGF-β1 was proposed to contribute crucially to maintenance of CML LSCs [99, 170] but its cellular source has not been determined. We proved that senescent megakaryocytes were a major source of TGF-β1 and demonstrated that p16- and p21-double-deficient LSCs failed to increase megakaryocyte numbers at the first transplantation and lacked the leukemogenic capability to cause CML development at the secondary transplantation [164]. We further revealed that bone marrow megakaryocytes in human CML patients expressed both p16 and p21, suggestive of senescence in these cells. Thus, it is likely that CML leukemia non-stem cells, BCR-ABL-transformed megakaryocytes, can support the leukemogenic capacity of CML LSCs by providing them with TGF-β1 (Fig. 6). Moreover, as the resistance to TKIs was well correlated with bone marrow megakaryocyte numbers at diagnosis, it is highly likely that megakaryocytes can contribute to LSC maintenance in human CML patients.

## Perspective on the roles of cancer non-stem cells in solid tumors

CSCs are presently presumed to be crucially involved in malignant progression of solid cancer: chemoresistance, radioresistance, immune evasion, and metastasis. Apparent morphological differences enabled us to identify non-stem cells such as basophils and megakaryocytes in CML. Basophil-derived CCL3 favors LSC-mediated hematopoiesis by suppressing normal hematopoiesis while megakaryocyte-derived TGF-β1 maintains the stemness of LSCs. Thus, leukemia non-stem cells have indispensable roles in the proliferation and maintenance of LSCs in CML. A similar observation was observed on glioblastoma, a representative solid cancer, which arises from CSCs [9, 10]. Bastola and colleagues demonstrated that CSCs were enriched at tumor edge compared to tumor core sites of GBM tissues and that the cells at core sites released soluble CD109 to induce CSCs at tumor edge to proliferate and to display radioresistance [171]. Thus, even in solid cancer, cancer non-stem cells may contribute to cancer development and progression as in the case of CML. However, more elaborate methods are required to be developed to discriminate precisely cancer non-stem cells from CSCs, for extensive clarification on the roles of cancer non-stem cells in cancer development and progression.

## Data Availability

Not applicable.

## Abbreviations

ABC: ATP-binding cassette
ABCB1: ABC subfamily-B member 1
ABCG2: ABC subfamily-G member 2
AP: Accelerated phase
ALDH: Aldehyde dehydrogenase
AML: Acute myeloid leukemia
ATM: Ataxia-telangiectasia-mutated
Bcl-2: B-cell lymphoma 2
BC: Blast crisis
bFGF: Basic fibroblast growth factor
BMP: Bone morphogenic protein
CAF: Cancer-associated fibroblast
CD44s: Standard form of CD44
CD44v: Variant CD44 isoform
CEBP: CCAAT/enhancer binding protein
CFU: Colony-forming unit
ch: Chromosome
CML: Chronic myeloid leukemia
CP: Chronic phase
CSC: Cancer stem cell
DPP: Dipeptidyl peptidase
EMT: Epithelial-mesenchymal transition
GM: Granulocyte-macrophage
HGF: Hepatocyte growth factor
HIF: Hypoxia inducible factor
HPC: Hematopoietic progenitor cell
HSC: Hematopoietic stem cell
HSPC: Hematopoietic stem/progenitor cell
IL: Interleukin
JAK: Janus kinase
LSC: Leukemia stem cell
MIP: Macrophage inflammatory protein
NF-κB: Nuclear factor- κB
NK: Natural killer
PI3K: Phosphoinositide 3-kinase
ROS: Reactive oxygen species
SASP: Senescence-associated secretory phenotype
SP: Side population
SR-B2: Scavenger receptor-B2
STAT: Signal transducer and activator of transcription
TGF: Transforming growth factor
TKI: Tyrosine kinase inhibitor
Treg: Regulatory T cell
TSLP: Thymic stromal lymphopoietin
VEGF: Vascular endothelial growth factor
